# Surprising abundance of *Gallionella*-related iron oxidizers in creek sediments at pH 4.4 or at high heavy metal concentrations

**DOI:** 10.3389/fmicb.2013.00390

**Published:** 2013-12-18

**Authors:** Maria Fabisch, Felix Beulig, Denise M. Akob, Kirsten Küsel

**Affiliations:** ^1^Aquatic Geomicrobiology Group, Institute of Ecology, Friedrich Schiller University JenaJena, Germany; ^2^U.S. Geological Survey, National Research ProgramReston, VA, USA

**Keywords:** acid mine drainage, slightly acidic, metal tolerance, microbial ecology, bacterial community structure, *Gallionella* spp., *Sideroxydans* spp.

## Abstract

We identified and quantified abundant iron-oxidizing bacteria (FeOB) at three iron-rich, metal-contaminated creek sites with increasing sediment pH from extremely acidic (R1, pH 2.7), to moderately acidic (R2, pH 4.4), to slightly acidic (R3, pH 6.3) in a former uranium-mining district. The geochemical parameters showed little variations over the 1.5 year study period. The highest metal concentrations found in creek sediments always coincided with the lowest metal concentrations in creek water at the slightly acidic site R3. Sequential extractions of R3 sediment revealed large portions of heavy metals (Ni, Cu, Zn, Pb, U) bound to the iron oxide fraction. Light microscopy of glass slides exposed in creeks detected twisted stalks characteristic of microaerobic FeOB of the family *Gallionellaceae* at R3 but also at the acidic site R2. Sequences related to FeOB such as *Gallionella ferruginea, Sideroxydans* sp. CL21, *Ferritrophicum radicicola*, and *Acidovorax* sp. BrG1 were identified in the sediments. The highest fraction of clone sequences similar to the acidophilic “*Ferrovum myxofaciens*” was detected in R1. Quantitative PCR using primer sets specific for *Gallionella* spp., *Sideroxydans* spp., and “*Ferrovum myxofaciens*” revealed that ~72% (R2 sediment) and 37% (R3 sediment) of total bacterial 16S rRNA gene copies could be assigned to groups of FeOB with dominance of microaerobic *Gallionella* spp. at both sites. *Gallionella* spp. had similar and very high absolute and relative gene copy numbers in both sediment communities. Thus, *Gallionella*-like organisms appear to exhibit a greater acid and metal tolerance than shown before. Microaerobic FeOB from R3 creek sediment enriched in newly developed metal gradient tubes tolerated metal concentrations of 35 mM Co, 24 mM Ni, and 1.3 mM Cd, higher than those in sediments. Our results will extend the limited knowledge of FeOB at contaminated, moderately to slightly acidic environments.

## Introduction

Microbial iron oxidation is mediated by iron-oxidizing bacteria (FeOB), which obtain energy for growth from Fe(II) oxidation (Konhauser, 2007) and facilitate the precipitation of Fe(III) oxyhydroxides. Microbial Fe(II) oxidation occurs from acidic to neutral pH (Kappler and Straub, 2005). At extremely acidic pH, Fe(II) is stable in the presence of oxygen due to a very low iron oxidation rate allowing acidophilic FeOB to oxidize Fe(II) under oxic conditions. At higher pH the rate of chemical Fe(II) oxidation increases with the most rapid rate occurring at neutral pH (Stumm and Morgan, 1996). Thus, under circumneutral conditions FeOB have to compete with chemical iron oxidation and thrive in microoxic or anoxic environmental niches (Ehrenreich and Widdel, 1994; Straub et al., 1996; Emerson and Moyer, 1997).

Iron redox processes are important at acidic, acid mine drainage (AMD)-impacted sites, because iron is often one of the most abundant metals and other heavy metals can adsorb to or co-precipitate with Fe(III) precipitates (Stumm and Morgan, 1996). Microbial Fe(II) oxidation can promote metal(loid) retardation via formation of Fe(III) oxide precipitates (Fukushi et al., 2003; Hohmann et al., 2010). Microbial iron oxidation has been well studied in a number of extremely AMD locations, such as the Richmond Mine system, Iron Mountain, CA (Druschel et al., 2004), and abandoned mines in North Wales (Hallberg et al., 2006). Microbial communities in these extremely acidic environments are characterized by a few dominant groups (Baker and Banfield, 2003; Lear et al., 2009), e.g., the well-known acidophilic, aerobic FeOB *Acidithiobacillus ferrooxidans, Leptospirillum ferrooxidans*, or “*Ferrovum myxofaciens*” (Hallberg, 2010). Acidophilic FeOB are known to tolerate heavy metal concentrations up to the mM to M range (Dopson et al., 2003; Watkin et al., 2009). In contrast, most neutrophilic FeOB did not originate from contaminated environments and their metal tolerances have not been studied.

Neutrophilic FeOB are phylogenetically and metabolically diverse, oxidizing iron under microoxic (microaerobic FeOB) or anoxic conditions (nitrate-reducing or phototrophic FeOB) (e.g., Ehrenreich and Widdel, 1994; Hafenbradl et al., 1996; Emerson and Moyer, 1997; Sobolev and Roden, 2004; Weiss et al., 2007). Numerous neutrophilic FeOB belong to the *Proteobacteria* (Hedrich et al., 2011), such as the microaerobic *Leptothrix* spp., *Gallionella* spp., and *Sideroxydans* spp., or the nitrate-dependent *Thiobacillus denitrificans* and *Acidovorax* spp. However, less is known about microbial iron oxidation at moderately acidic pH (pH 4–6). The microaerobic strain *Sideroxydans* sp. CL21 can oxidize ferrous iron between pH 4 and 6, the microaerobic FeOB *Ferritrophicum radicicola* can grow in a pH range of 4.5–7, and *Gallionella* spp. are known to grow at pH 5–7.5 (Hallbeck and Pedersen, 1990; Emerson and Moyer, 1997; Hanert, 2006; Weiss et al., 2007; Lüdecke et al., 2010). To our knowledge there are only few studies on slightly acidic sites with high metal load and iron-oxidizing/-reducing organisms, e.g., Hallberg and Johnson (2003), Sanchez-Andrea et al. (2012).

Given the slower rates of abiotic Fe(II) oxidation in moderately acidic environments compared to circumneutral ones, FeOB may be also important in the bioremediation of moderately acidic AMD waters and environments (Hallberg and Johnson, 2003). Therefore, it is important to have a detailed understanding of the geochemistry and microbial populations involved in iron redox reactions at AMD sites, especially at different pH values that occur due to downstream migration of AMD. We expect a shift in the bacterial FeOB community structure at AMD sites with increasing pH, from acid- and heavy metal-tolerant to neutrophilic FeOB that might be less metal tolerant. Given the faster abiotic Fe(II) oxidation at pH > 5, FeOB should be less abundant at slightly acidic sites especially with high heavy metal loads, which might be toxic to FeOB.

Metal-contaminated creeks in the former uranium-mining district of Ronneburg, Germany, cover a wide pH range from extremely (pH 2.7) to slightly acidic conditions (pH 6.3) and thus, provide a valuable environment to study the effect of differing pH on FeOB. Thus, we (i) elucidated the distribution of heavy metals in creek water and its corresponding sediments over a 1.5 year period, (ii) identified and quantified FeOB in creek sediments using clone libraries and quantitative PCR (qPCR) with specific primer sets, and (iii) tested the metal tolerance of Fe(II)-oxidizing enrichment cultures. Surprisingly, the total abundance of FeOB was not dependent on pH or the heavy metal load, nor was the dominance of the neutrophilic *Gallionella*-related organisms.

## Materials and methods

### Field site and sampling

The three study sites R1, R2, and R3 are located near the former heaps of the former Ronneburg uranium-mining district (Thuringia, Germany; Supplementary Figure S1). Site R2 and R3 are located in the former creek bed of the Gessenbach creek, while R1 is located at the afflux of a drainage creek to a seepage water collection basin. The area is contaminated due to former acid leaching of low-grade black shale, and despite physical remediation in the 1990s groundwater and creeks are severely contaminated with heavy metals (Jakubick et al., 1997). All three creek sites are only spring-fed by highly heavy metal-contaminated ground and seepage waters. At numerous time points from December 2008 to May 2010, sediment pH and redox potential, as well as creek water pH, redox potential, and dissolved oxygen were measured *in situ* with respective electrodes and meters (Mettler Toledo; WTW; sediment: put meters ca. 2 cm directly in). Redox potential was corrected to the standard hydrogen electrode. Collected water and sediment samples were transported and stored anoxically at 4°C until further processing (within 24 h). Water was filtered (0.45 μm, PVDF), then stored at −20°C for nitrate analysis or acidified and stored at 4°C for dissolved organic carbon (DOC), sulfate, and metal analyses. Sediment samples were taken from the upper 5 cm (site R1, R2) or 10–15 cm (site R3) of three different spots per site and pooled for chemical analyses. Cores (diameter ca. 6 cm) were obtained from R3 sediment for oxygen profiles and transported to the laboratory at 4°C with an overlying creek water column. Sediment samples for specific experiments were taken aseptically.

### Geochemical characterization of creek water and sediment

Fresh triplicate sediment samples were dried at 60°C for 3 days to determine dry weights (wt) and after milling (Mixer Mill MM301, Retsch, Germany) were used to measure total organic carbon (TOC) with a CN analyzer (vario MAX CN, Elementar, Germany). DOC in water was measured by catalytic combustion oxidation using a TOC analyzer (TOC-VCPN, Shimadzu, Germany). Triplicate oxygen profiles in a R3 sediment core taken in June 2009 were performed with a microelectrode (OX-100, connected to picoammeter PA2000, Unisense, Aarhus, Denmark). Sulfate and nitrate were analyzed in triplicate using the barium chloride method (Tabatabai, 1974) and resorcin method (Velghe and Claeys, 1985), respectively. Creek sediment was extracted as needed to determine water-solvable sulfate and KCl-extractable nitrate (Forster, 1995). Dissolved heavy metals (Fe, Cu, Ni, Zn, Cd) in creek water were measured using inductively coupled plasma mass spectrometry (ICP-MS; X-Series II, Quadrupol, Thermo Electron, Germany). Total heavy metal contents of dried (60°C for 3 days) and milled (Mixer Mill MM301, Retsch, Germany) sediments as well as of sediment fractions after sequential extraction were analyzed after total acid digestion by ICP-MS or ICP-optical emission spectrometry (Spectroflame ICP-OES, Spectro Analytical Instruments Inc., Germany). Sequential extraction of freeze-dried R3 sediment (field triplicates taken in July 2011 and pooled after drying) was done according to Grawunder et al. (2009). Fe(II) was determined in triplicate samples with the phenanthroline method (Tamura et al., 1974) after extraction in 0.5 N HCl for 1 h at 22°C. HCl extractable Fe(III) in creek water was calculated from the increase in Fe(II) concentration after the addition of ascorbic acid (0.6% final concentration). Fe(III) in creek sediment was calculated as difference between total Fe, measured by ICP-OES, and HCl-extractable Fe(II).

### Enumeration of microaerobic and nitrate-reducing FeOB

Culturable microaerobic and nitrate-reducing FeOB were enumerated in all creek sediments (obtained in December 2008) using a most probable number (MPN) technique (De Man, 1975) using 4-fold serial dilutions in sterile 0.7% NaCl with three replicates. Microaerobic FeOB were grown in gradient tube cultures (Emerson and Moyer, 1997) modified as described by Lüdecke et al. (2010), with a FeS plug and a semi-solid overlayer. Nitrate-reducing FeOB were cultivated in an anoxic bicarbonate-buffered mineral medium [modified after Widdel and Bak (1992); L^−1^: NaCl, 1 g; MgCl_2_ * 6H_2_O, 0.4 g; CaCl_2_ * 2H_2_O, 0.1 g; NH_4_Cl, 0.3 g; KH_2_PO_4_, 0.34 g; KCl, 0.5 g; 1 M NaHCO_3_, 30 mL; vitamin solution (Balch et al., 1979), 1 mL; trace element solution SL 9 (Tschech and Pfennig, 1984), 1 mL; pH 7.0] containing 10 mM Fe(II) and 4 mM nitrate. To mimic environmental conditions, both media were amended with a sediment extract (1:60 or 1:200, respectively), which was obtained by diluting creek sediment 1:5 in ultra-pure water, thoroughly mixing for 10 min, centrifuging for 5 min at 2,700 × *g*, followed by sterile filtration of the supernatant. The sediment extract contained 26 mg L^−1^ DOC and heavy metals in the μM range. Cultures were incubated at 22°C for 24 days (microaerobic FeOB) or horizontally at 15°C for 10 weeks (nitrate-reducing FeOB) in the dark. Tubes were considered positive based on the formation of a distinct rust colored band compared to diffuse iron oxidation in uninoculated controls (microaerobic FeOB) or on consumption of nitrate and Fe(II) compared to uninoculated controls (nitrate-reducing FeOB). MPN values and 95% confidence limits were calculated from standard MPN tables (De Man, 1983).

### Metal tolerance of microaerobic FeOB from R3 sediment

To evaluate Co, Ni, or Cd tolerance of enriched microaerobic FeOB, we modified the media used in the gradient tubes. To prevent metal precipitation, as it was observed after addition of metals to the overlayer, two overlayers were applied in Co- and Ni-amended tubes: First 1 ml of overlayer medium without metals was added to the FeS plug and after solidification 5 ml of Co- or Ni-containing overlayer medium was added on top. Cd-amended tubes were prepared with a single overlayer containing Cd, but with 30 mM of FeSO_4_ in the plug instead of FeS. This was done because the two-overlayer-method still resulted in precipitation when Cd was added. Gradient tubes amended with 0.5, 1.0, 5.0, 10 or 50 mM of CoCl_2_, NiCl_2_, or CdCl_2_ were inoculated with a 10^−1^ dilution of R3 sediment in 0.7% NaCl (4-fold with two uninoculated controls each) and incubated for 6 weeks. Dissolved metal concentrations were measured by ICP-MS to check for interference of media components with Co, Ni or Cd in the overlayer (Supplementary Table S1): The overlayer of uninoculated controls was collected within 24 h of preparation, centrifuged for 10 min at 3,000 × *g*, and the supernatant was filtered through a 0.45 μM pre-filter followed by a 0.2 μM filter to remove precipitates and agarose. Samples were acidified with nitric acid and stored at 4°C until analysis.

### Light microscopy

Duplicate glass slides protected by perforated 50 mL plastic tubes were exposed at all three sites into creek sediment (ca. 0–10 cm depth) or water for 1 or 2 weeks (after Hanert, 2006). Slides were transported and stored in 0.7% NaCl solution at 4°C and examined by light microscopy (Axioplan universal microscope, Zeiss, Germany).

### Clone library construction and statistical analysis

DNA and RNA were extracted from creek sediments (pooled material from triplicate field samples obtained in May 2010) using the PowerSoil DNA or Total RNA Isolation Kit (MO BIO Laboratories, Inc., CA, USA). Residual DNA was removed from RNA extracts with RNase-free DNase I (Fermentas, Germany) following the manufacturer's protocol. RevertAid Reverse Transcriptase and Random Hexamer Primer (Fermentas, Germany) were used to produce cDNA. DNA or cDNA were PCR amplified using *Bacteria* domain-specific 16S rRNA gene primers fD1 and rP2 (Weisburg et al., 1991). DNA contamination of RNA extracts was monitored by PCR amplification of DNase-treated RNA without reverse-transcription; no contaminating DNA was detected. Purified (NucleoSpin Extract II, Macherey-Nagel, Germany) PCR products were cloned using the pGEM-T Easy vector according to the manufacturer's protocol (Promega, USA) and then sequenced (Macrogen, Seoul, South Korea) using the 16S rRNA gene primer 907R (Muyzer et al., 1995). Sequences were trimmed using Geneious Pro v. 4.6.0 (Drummond et al., 2009) and nearest relatives were identified using BLAST against the NCBI GenBank database (Johnson et al., 2008). Sequences were grouped into operational taxonomic units (OTUs) based on a 97% sequence cut-off. Rarefaction analysis (Heck et al., 1975; Holland, 2003) was assessed using Analytic Rarefaction 2.0. Coverage was calculated as described by Singleton et al. (2001).

### Quantitative pcr (qPCR)

Copy numbers of 16S rRNA genes in creek sediments were determined by qPCR with group-specific primers for *Bacteria, Archaea*, and known FeOB- and iron-reducing bacteria (FeRB)-related groups: *Gallionella* spp., *Sideroxydans* spp., and “*Ferrovum myxofaciens*” (FeOB), as well as *Albidoferax ferrireducens, Geobacter* spp., and *Acidiphilium* spp. (FeRB). For this, genomic DNA was extracted from R2 and R3 sediments (obtained in February 2009) as described above. DNA extraction from R1 sediment was not successful. Aliquots of 1–10 ng of DNA were used in triplicates as templates for qPCR on a Mx3000P real-time PCR system (Stratagene, USA) with the Maxima SYBR Green qPCR Mastermix kit (Fermentas, Canada). Standard curves were prepared by serial dilutions of different plasmids for the different organism groups, each containing a sequence affiliated with the respective group. Melting curve analysis was used to confirm the specificities of qPCR products. PCR grade water and TE buffer were always included as non-template controls.

Used primers and standard plasmids as well as qPCR conditions were given in Herrmann et al. (2012) for *Archaea* and in Lu et al. (2013) for *Bacteria*, “*Ferrovum myxofaciens*,” *Albidoferax ferrireducens, Geobacter* spp., and *Acidiphilium* spp. *Gallionella*-related 16S rRNA gene copy numbers were determined with primers 122f and 384r (Heinzel et al., 2009b) and a plasmid as standard, which contained a *Gallionella*-related 16S rRNA gene sequence obtained from an iron ochre encrusted groundwater well (provided from J. Wang, GenBank accession number JX855939). The *Gallionella* qPCR was run with 50 cycles, with denaturation at 95°C for 15 s, annealing at 55°C for 20 s, extension at 72°C for 25 s, and amplification fluorescence collection at 78°C for 10 s.

*Sideroxydans*-related 16S rRNA gene copy numbers were determined with newly designed primers Sid-120F (5′-TGT ATC GGA ACA TGT CCG GA-3′) and Sid-467R (5′- CCG TCA TCC ATA CAG AGT-3′). Primers were designed to target the 16S rRNA gene sequences of *Sideroxydans* spp. using the ARB software package (Ludwig et al., 2004). Specificity was confirmed beforehand using online probe match databases (Johnson et al., 2008; Loy et al., 2008; Cole et al., 2009) as well as PCR and qPCR verification. For the PCR and qPCR we used DNA from *Sideroxydans* strains ES-1 and CL21 or from *Gallionellaceae* strain HDD as a template, or plasmids containing cloned sequences related to various organisms within the *Proteobacteria* (*Sideroxydans* sp. CL21, *Gallionella* spp., *Thiobacillus* sp., *Dechloromonas* sp., *Albidoferax ferrireducens, Xanthomonadaceae, Toluomonas* sp., *Acidocella* sp., *Pleomorphomonas* sp., *Geobacter* sp.). The specificity of the newly designed *Sideroxydans* spp. primer pair was further verified by constructing a clone library (33 clones) with PCR products, which were amplified from R2 sediment DNA extract with the qPCR primers using the same cycling conditions as in the qPCR. Clone library construction was conducted as discribed above, except sequencing was done with the plasmid-specific primer SP6 (5′-ATT TAG GTG ACA CTA TAG-3′). As a standard we used a plasmid containing a *Sideroxydans* sp. CL21-related 16S rRNA gene sequence obtained from Gessenbach creek water (99% sequence identity, GenBank accession number KF424870). The *Sideroxydans* qPCR was run with 50 cycles, with denaturation at 95°C for 30 s, annealing at 56°C for 30 s, extension at 72°C for 50 s, and amplification fluorescence collection at 78°C for 15 s. Standard curves were linear from 5 × 10^7^ to 5 × 10^2^ copies with R^2^ values of 0.999–1.000 and the qPCR performed with efficiencies ranging from 86 to 90%.

16S rRNA gene copy numbers were used to calculate the estimated percentage of the bacterial community of the single FeOM-/FeRM-groups by including the specific number of 16S rRNA genes per genome in the respective organism group. The number of 16S rRNA genes per genome was obtained from the rrnDB database version 3.1.221 (Lee et al., 2009), if available (*Bacteria*, 4.2; *Gallionella* spp., 3; *Albidoferax ferrireducens*, 2; *Geobacter* spp., 2.4; *Acidiphilium* spp., 2). For *Sideroxydans* spp. the value of two 16S rRNA genes per genome was used based on one published genome (*Sideroxydans* sp. ES-1; GenBank accession CP001965). For “*Ferrovum myxofaciens*” the value of one 16S rRNA gene per genome was used (Sophie Mosler, personal communication).

### Nucleotide sequence accession numbers

The representative 16S rRNA and 16S rRNA gene OTU sequences generated in clone libraries in this study were deposited in the GenBank database under the accession numbers JN885797 to JN885880.

## Results

### Creek geochemistry

**Figure 1:**
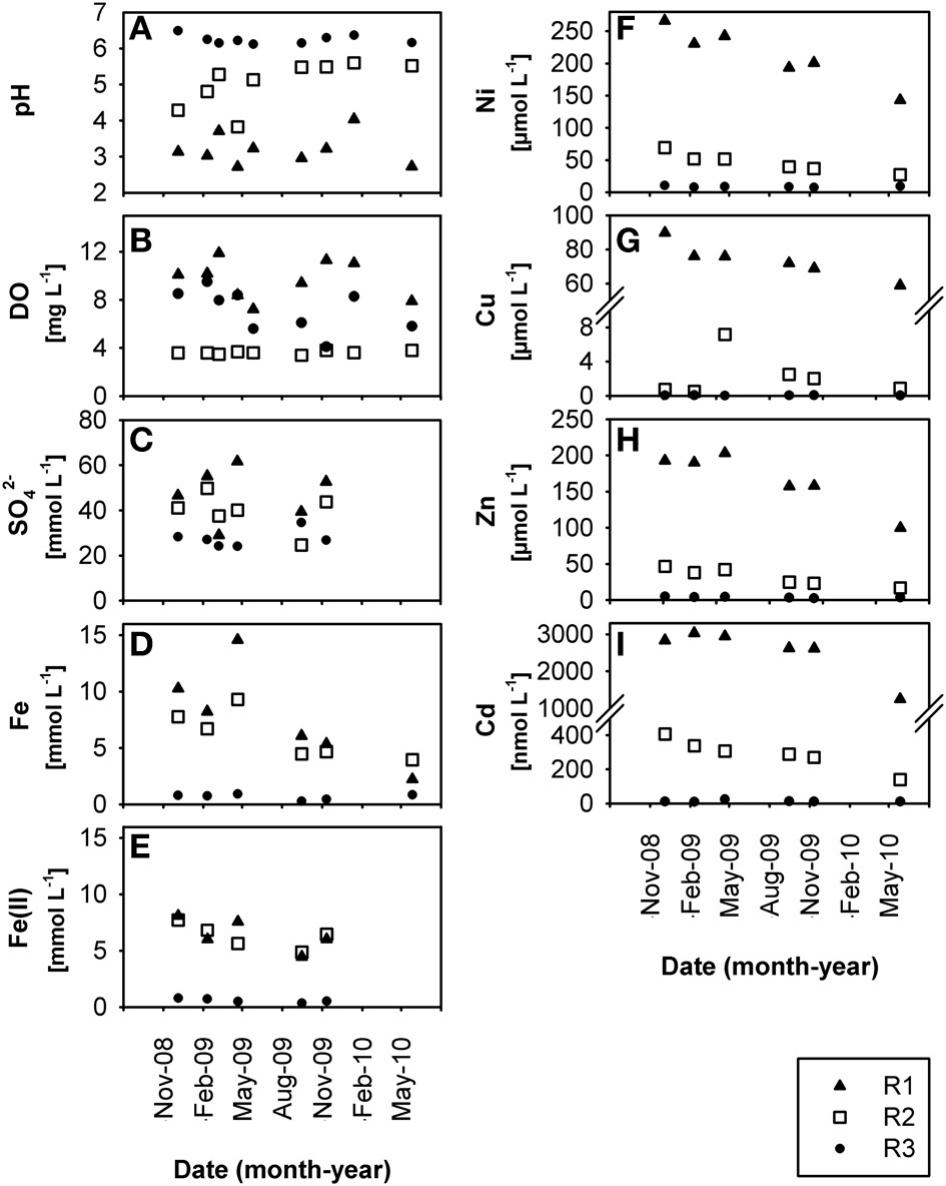
**Time-resolved chemical data for creek water: (A)** pH, **(B)** dissolved oxygen, DO, **(C)** dissolved sulfate, SO^2−^_4_, **(D)** dissolved total Fe, **(E)** dissolved HCl-extractable Fe(II), **(F)** dissolved Ni, **(G)** dissolved Cu, **(H)** dissolved Zn, **(I)** dissolved Cd, at sites R1, R2, and R3 in the former Ronneburg uranium-mining district, Germany.

**Figure 2:**
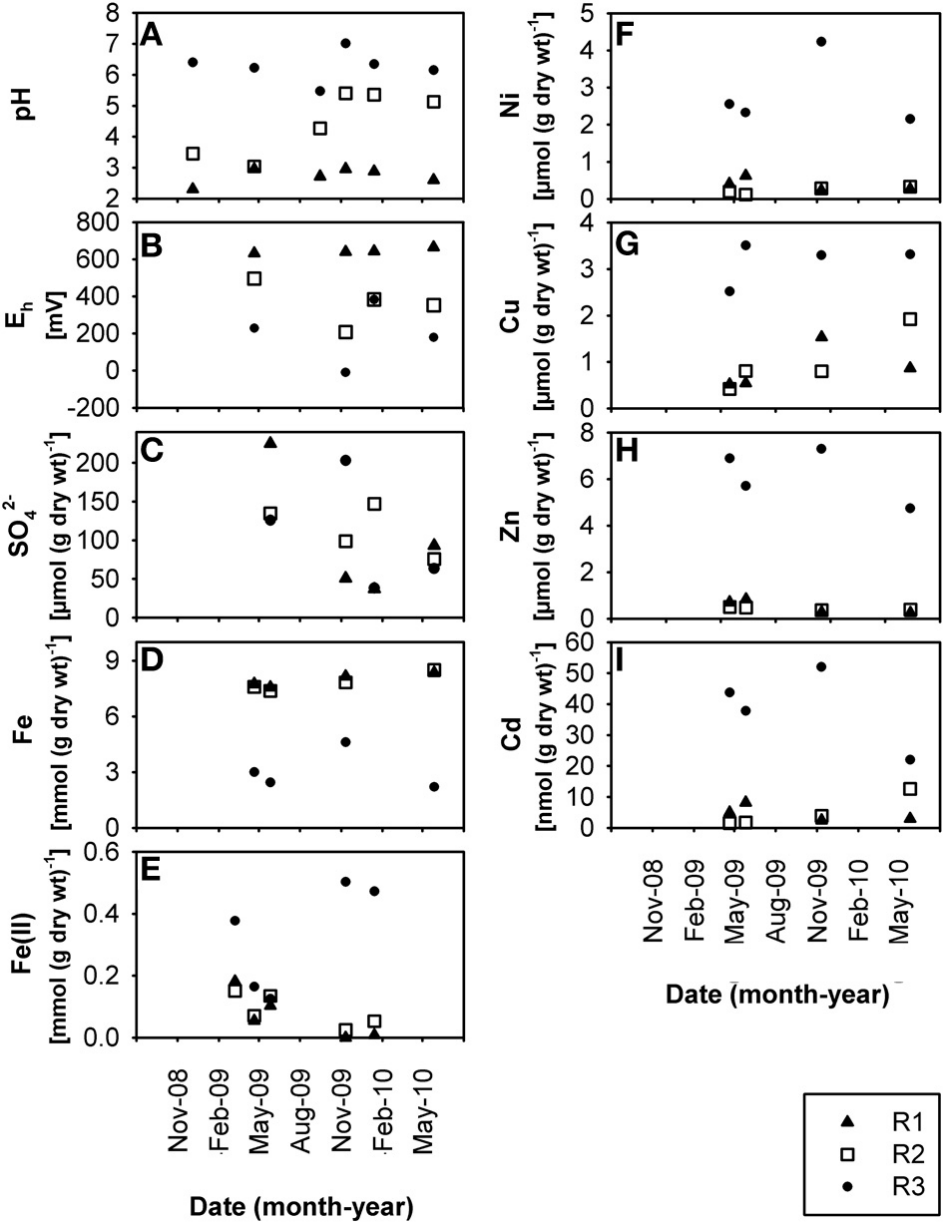
**Time-resolved chemical data for creek sediment: (A)** pH, **(B)** redox potential, E_h_, **(C)** sulfate, SO^2−^_4_, **(D)** total Fe content, **(E)** HCl-extractable Fe(II)content, **(F)** total Ni content, **(G)** total Cu content, **(H)** total Zn content, **(I)** total Cd content, at sites R1, R2, and R3. wt, weight.

The three creek sites R1, R2, and R3 (Supplementary Figure S1) within the former uranium-mining area near Ronneburg, Germany, showed differences in pH and heavy metal loads in waters and sediments. Creek sites R1 and R2 were extremely acidic (average R1 sediment pH 2.7 ± 0.3) and acidic (average R2 sediment pH 4.4 ± 1.0), respectively, with some variations in the pH at site R2 (Figures 1A, 2A) during the 1.5 year study period. R3 sediment had a stable, slightly acidic pH (average 6.3 ± 0.5; Figure 2A) and high heavy metal contamination, e.g., Ni, Cu, and Zn (average values 3.2, 2.8, and 6.2 μmol per g dry wt, respectively; Figures 2F–H). R3 sediment showed ~10-fold higher heavy metal concentrations than R1 and R2 sediments (Figures 2F–I), whereas creek water of site R3 had the lowest concentrations of dissolved heavy metals (0.05–20% of values of sites R1 and R2; Figures 1F–I). Time patterns of total Fe, Ni, Zn, and Cd in R3 sediment were similar to each other (Figures 2D,F,H,I). Sequential extraction of R3 sediment revealed that a large portion of the metals was bound to the amorphous and crystalline iron oxide fractions (Ni, 37%; Cu, 39%; Zn, 43%; Cd, 27%).

Creek site R3 had deep sediment (~0.5 m) compared to the thin R1 and R2 sediments (~0.1 m) with huge variations of the redox potential over time between −9 and +382 mV in sediment from the upper 5 cm (Figure 2B). Microelectrode measurements in a R3 sediment core located the oxic-anoxic transition zone within 3 mm sediment depth (data not shown), demonstrating a thick anoxic zone. The relative Fe(II) content in R3 sediment was higher (7.2% of total iron; Figures 2D,E, note axis) compared to R1 and R2 sediments (0.7% and 1.0% of total iron, respectively). Dissolved Fe(II) was still present in creek water of the pH 6.3 site R3 (average 0.7 ± 0.3 mmol L^−1^; Figure 1E), indicating that abiotic and biotic Fe(II) oxidation was possible. Dissolved Fe(III) in creek water was, as expected, only present at the extremely acidic site R1 (Figures 1D,E).

Low dissolved oxygen in creek water was found at site R2 (Figure 1B), reflecting the influence of a local drainage pipe (inflow with 1.7 mg oxygen L^−1^). Sulfate was present in high amounts at all sites in waters (ca. 20–60 mmol L^−1^; Figure 1C) and sediments (ca. 40–230 μmol per g dry wt; Figure 2C) due to acid leaching during mining. Nitrate had lower concentrations in water (R1, 165 ± 65; R2, 60 ± 42; R3, 93 ± 70 μmol L^−1^) and sediments (R1, 4.9 ± 3.9; R2, 0.8 ± 0.6; R3, 1.6 ± 1.3 μmol per g dry wt). DOC in creek waters was between 4.9 and 7.8 mg L^−1^. TOC in creek sediments was 0.7 ± 0.1% (R1), 1.0 ± 0.1% (R2), and 3.9 ± 0.3% (R3) of sediment dry wt.

### Presence and abundance of FeOB and FeRB

The abundance of cultured microaerobic FeOB in the sediments were similar to each other, within a range of 10^4^–10^5^ cells per g wet wt (R1, 0.2-4.8 × 10^4^; R2, 0.2-4.8 × 10^5^; R3, 0.4-8.9 × 10^4^ cells per g wet wt; values represent the ranges of MPN values within 95% certainty). Culturable nitrate-reducing FeOB were not detectable in R1 sediment, but had low abundance in R2 sediment (0.2-16.6 cells per g wet wt) and R3 sediment (0.5-9.3 × 10^2^ cells per g wet wt).

Light microscopy of glass slides exposed in creek sediment or water revealed the occurrence of numerous twisted stalks at sites R3 (pH 6.3) and R2 (pH 4.4), which were mostly associated with iron oxides (Supplementary Figure S2). Such stalks are characteristic of microaerobic FeOB such as the *Gallionellaceae* bacterium R-1 and *Gallionella ferruginea* (Hanert, 2006; Krepski et al., 2012) or the marine genus *Mariprofundus* (Emerson et al., 2007). These stalks were not observed at the pH 2.7 site R1.

**Figure 3:**
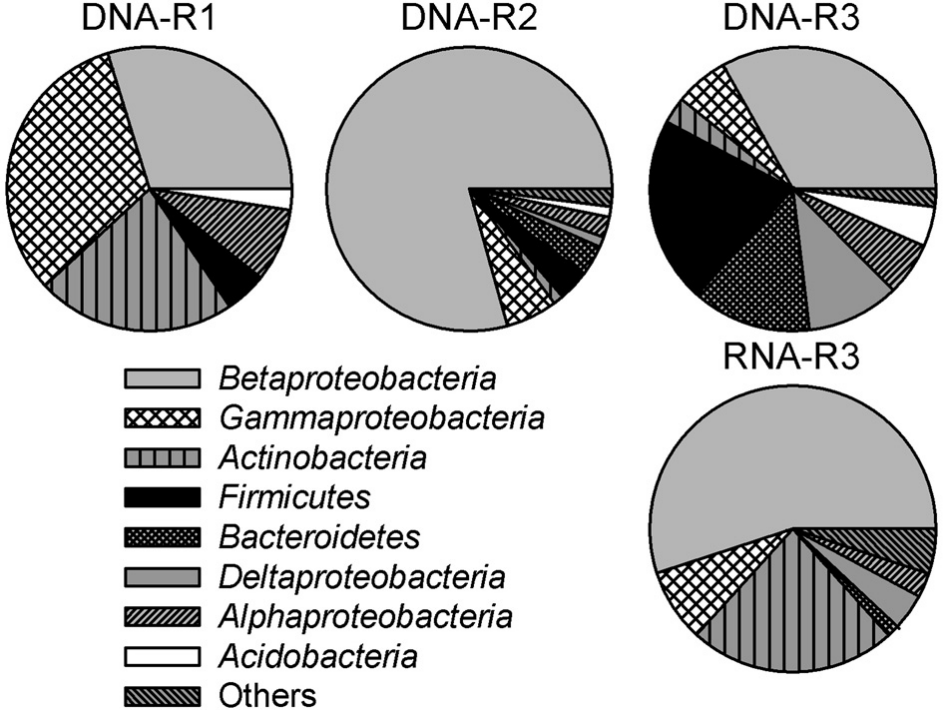
**Bacterial community composition in 16S rRNA (gene) clone libraries derived from creek sediment of sites R1 (DNA-R1), R2 (DNA-R2), and R3 (DNA-R3 and RNA-R3) in the former Ronneburg uranium-mining district, Germany.** Calculations were based on the total number of clones associated with phylotypes of sequenced representatives at the phylum level, or class level for *Proteobacteria*. The category “others” includes the low abundance groups *Chloroflexi, Epsilonproteobacteria, Gemmatimonadetes, Cyanobacteria, Nitrospirae, Planctomycetes, Synergistetes*, and the candidate phylum OP11.

To identify the most abundant phylogenetic groups potentially involved in iron oxidation (and reduction) in creek sediments, DNA- (R1, R2, R3) and RNA- (R3) derived 16S rRNA gene clone libraries were constructed. A total of 311 clone sequences (ca. 500–900 bp) were screened yielding 27, 19, 34, and 30 OTUs for libraries DNA-R1, DNA-R2, DNA-R3, and RNA-R3, respectively (Supplementary Table S2). Rarefaction analysis did not indicate full saturation (data not shown). Coverage was between 63% (DNA-R3) and 87% (DNA-R1) (Supplementary Table S2). The majority of clone sequences from the four libraries were affiliated with the *Proteobacteria* (classes *Alpha*-, *Beta*-, *Gamma*-, and *Deltaproteobacteria*), *Actinobacteria, Firmicutes*, and *Bacteroidetes* lineages, but the proportion differed between sites (Figure 3).

High fractions of clones (47% of all clones) derived from DNA and RNA extracts of pooled field samples showed ≥97% 16S rRNA gene sequence identity to reported FeOB or FeRB (Table 1). The relative fraction of FeOB-related clone sequences was highest in clone libraries DNA-R2 (73%) and RNA-R3 (28%). Clone sequences similar to reported FeOB (and FeRB) were found in higher fractions in RNA-R3 than in DNA-R3. The highest fraction of clone sequences similar to acidophilic FeOB/FeRB were detected in DNA-R1 representing sequences similar to the FeOB “*Ferrovum myxofaciens*” (Hallberg et al., 2006) and the FeRB *Acidiphilium acidophilum* (Shelobolina et al., 2007), which was only detected in this library (Table 1). The known neutrophilic, microaerobic FeOB *Gallionella ferruginea* (Hallbeck and Pedersen, 1990; Hanert, 2006) represented 6% of sequences in DNA-R1, 69% in DNA-R2, and 9% in DNA-R3; no other organism was detected with such dominance within a clone library. The dominant FeOB/FeRB-OTUs in the library RNA-R3 were related to *Acidovorax* sp. strain BrG1, a reported neutrophilic nitrate-reducing FeOB (Straub et al., 2004), and *Albidoferax ferrireducens*, a reported neutrophilic psychrotolerant FeRB (Finneran et al., 2003), which was not found in DNA-R3. Other FeOB detected in clone libraries were the microaerobic *Sideroxydans* sp. CL21 (Lüdecke et al., 2010) and *Ferritrophicum radicicola* (Weiss et al., 2007). Sequences related to *Stenotrophomonas maltophilia* strain BK, which is reported to reduce Fe(III) using xenobiotics as energy and carbon source (Ivanov et al., 2005), were detected at sites R1 and R2. But *Stenotrophomonas maltophilia* is also known as a laboratory contaminant in clone libraries (Tanner et al., 1998; Zehr et al., 2003).

**Table 1:** **Fractions of clone sequences with ≥97% sequence identity to reported FeOB or FeRB in 16S rRNA gene and 16S rRNA clone libraries constructed from creek sediment of sites R1 (DNA-R1), R2 (DNA-R2), and R3 (DNA-R3 and RNA-R3), as well as pH tolerance and environmental occurrence of the known microorganisms**.

**Organism (accession^1^; affiliation; sequence identity)**	**(pH 2.7)**	**(pH 4.4)**	**(pH 6.3)**		**pH tolerance^2^**	**Environmental occurrence^3^**
	**DNA-R1**	**DNA-R2**	**DNA-R3**	**RNA-R3**		
**FeOB**	**15%**	**73%**	**13%**	**28%**		
*Gallionella ferruginea* (L07897; β-*Proteobacteria*; 98%)	6%	69%	9%	0%	Neutrophilic (5.0–7.6)^4^	Freshwater (springs, wells, drainages, groundwater seep, river sediment)^5^; AMD^6^
*Acidovorax* sp. strain BrG1 (U51101; β-*Proteobacteria*; 98-99%)	2%	0%	4%	24%	Neutrophilic (6.7)^7^	Freshwater sediment^8^; U-contaminated sediment^9^
“*Ferrovum myxofaciens*” (HM044161; β-*Proteobacteria*; 97%)	6%	1%	0%	3%	Extremely acidophilic (above 2)^10^	AMD^11^
*Ferritrophicum radicicola* (DQ386263; β-*Proteobacteria*; 99%)	0%	3%	0%	1%	Moderately acidophilic (4.5-7.0)^12^	Wetland plant roots^12^; AMD^13^
*Sideroxydans* sp. strain CL21 (GU134935; β-*Proteobacteria*; 98%)	1%	0%	0%	0%	Moderately acidophilic (4.0–6.0)^14^	Minerotrophic fen^14^, acidic lakes^15^; metal-contaminated soil^16^
**FeRB**	**16%**	**7%**	**5%**	**25%**		
*Albidoferax ferrireducens*^17^ (AF435948; β-*Proteobacteria*; 98%)	5%	0%	0%	22%	Neutrophilic (6.7–7.1)^18^	Freshwater (lakes, ponds, glaciers, hot springs, groundwater, acidic lake)^18^,^19^; wastewater sludge^20^; marine water^21^
*Stenotrophomonas maltophilia* (AY641540; γ-*Proteobacteria*; 99%)	6%	5%	0%	0%	Slightly basophilic (7.2–8.1)^22^	Waste water treatment plant^22^
*Geobacter argillaceus* (DQ145534; δ-*Proteobacteria*; 97%)	0%	1%	5%	3%	Neutrophilic (5.8–7.4)^23^	Kaolin clay^23^; wetland sediment^24^
*Acidiphilium acidophilum*^25^ (NR_036837; α-*Proteobacteria*; 99%)	5%	0%	0%	0%	Acidophilic (1.5–6.0)^26^	AMD^27^
*Geothrix fermentans* (U41563; *Acidobacteria*; 97%)	0%	1%	0%	0%	Not determined^28^	Contaminated environments (aquifers or soils with hydrocarbons, petroleum, U)^28^,^29^
**FeOB + FeRB**	**31%**	**80%**	**18%**	**53%**		

1 GenBank accession number.

2 pH tolerance represents reported pH tolerance or assumed pH tolerance based on the reported pH range for iron oxidation or reduction. In parentheses, the pH range for growth is given.

3 Environmental occurrence refers to either the source of isolation or detection with molecular methods.

17 Previously known as Rhodoferax ferrireducens (Ramana and Sasikala, 2009).

25 Previously known as Thiobacillus acidophilus (Hiraishi et al., 1998).

References for iron oxidation, iron reduction, pH, and occurrence:

4 (Hallbeck and Pedersen, 1990; Hanert, 2006);

5 (Hanert, 2006; Bruun et al., 2010; Yu et al., 2010);

6 (Bruneel et al., 2006; He et al., 2007; Heinzel et al., 2009a; Lear et al., 2009);

7 (Straub et al., 2004);

8 (Buchholz-Cleven et al., 1997);

9 (Akob et al., 2007);

10 (Hallberg, 2010);

11 (Hallberg et al., 2006; Suto et al., 2007; Heinzel et al., 2009a,b; Tan et al., 2009; Ziegler et al., 2009; Hallberg, 2010; Brown et al., 2011; Bruneel et al., 2011; Gonzalez-Toril et al., 2011; Kimura et al., 2011);

12 (Weiss et al., 2007);

13 (Gonzalez-Toril et al., 2011);

14 (Lüdecke et al., 2010);

15 (Percent et al., 2008, GenBank EF520447); (Reiche et al., 2011, GenBank FR667790);

16 (Satchanska et al., 2004, GenBank AJ582038);

18 (Finneran et al., 2003);

19 (Eriksson et al., 2005; Allgaier and Grossart, 2006; Matsuzawa et al., 2010; Nishio et al., 2010; Garcia-Echauri et al., 2011; Reiche et al., 2011);

20 (Jin et al., 2011);

21 (Lindh et al., 2013);

22 (Ivanov et al., 2005);

23 (Shelobolina et al., 2007);

24 (Roden et al., 2010);

26 (Guay and Silver, 1975; Johnson and McGinness, 1991);

27 (Hiraishi et al., 1998; Peccia et al., 2000);

28 (Coates et al., 1999);

29 (Anderson et al., 1998; Brodie et al., 2006).

FeOB, iron-oxidizing bacteria. FeRB, iron-reducing bacteria. AMD, acid mine drainage.

**Figure 4:**
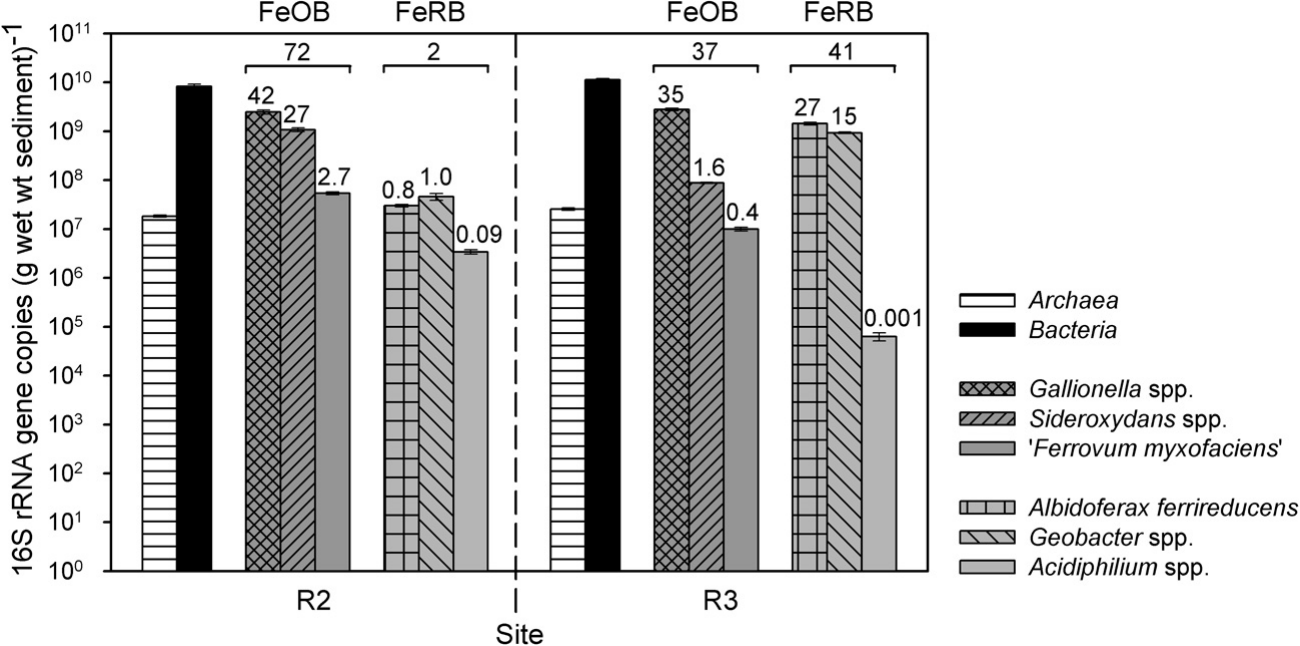
**16S rRNA gene copy numbers of different organism-groups per g wet wt sediment of sites R2 (pH 4.4) and R3 (pH 6.3).** Given are means and standard deviations of triplicate qPCR measurements with group-specific primers. The numbers above the bars represent the % of bacterial communities of a certain FeOB (*Gallionella* spp., *Sideroxydans* spp., “*Ferrovum myxofaciens*”) or FeRB (*Albidoferax ferrireducens, Geobacter* spp., *Acidiphilium* spp.) group, whereas the numbers above the brackets represent the sum % of bacterial communities of all three tested FeOB groups or all three tested FeRB groups together. wt, weight; FeOB, iron-oxidizing bacteria; FeRB, iron-reducing bacteria.

Group-specific qPCR with genomic DNA as template was used to estimate the relative abundance of known FeOB in R2 and R3 sediment. In addition, we also quantified FeRB as they might be important in the thick and more reduced R3 sediment. Total bacterial 16S rRNA gene copy numbers approximated 10^10^ copies per g wet wt sediment at sites R2 and R3; *Archaea* were much less abundant than *Bacteria* (Figure 4). The FeOB groups *Gallionella* spp., *Sideroxydans* spp., and “*Ferrovum myxofaciens*” together accounted for 72% (R2) and 37% (R3) of the bacterial community; while the FeRB groups *Albidoferax ferrireducens, Geobacter* spp., and *Acidiphilium* spp. together accounted for ca. 2% (R2) and 41% (R3) of the *Bacteria* (Figure 4). At site R2, *Gallionellaceae* were dominant with 2.5 × 10^9^ (*Gallionella*) and 1.1 × 10^9^ (*Sideroxydans*) gene copies per g wet wt sediment, representing ca. 42 and 27% of the total bacterial community, respectively. All other tested FeOB and FeRB groups comprised less than 3% of the bacterial community in R2 sediment. Three of the tested FeOB/FeRB groups accounted for more than 2% in R3 sediment: *Gallionella* (2.8 × 10^9^ gene copies per g wet wt; representing ca. 35% of the bacterial community), *Albidoferax* (1.4 × 10^9^ gene copies per g wet wt; ca. 27%), and *Geobacter* (9.3 × 10^8^ gene copies per g wet wt; ca. 15%) (Figure 4).

### Metal tolerance of microaerobic FeOB

Enriched microaerobic FeOB from the pH 6.3 R3 creek sediment grew in the newly developed metal gradient tubes amended with a maximum of 50 mM Co, 50 mM Ni, or 10 mM Cd (Supplementary Table S1); growth was indicated by the presence of a distinct rust-colored ring of iron oxides. The highest concentrations of dissolved metals where FeOB growth was observed were 34.45 mM Co, 24.01 mM Ni, and 1.32 mM Cd, as measured in uninoculated controls (Supplementary Table S1). It is likely that a proportion of the metals added to the medium sorbed to agarose or iron oxides formed or precipitated via reactions with the medium as was observed previously for FeRB medium (Burkhardt et al., 2011). Therefore, FeOB might not face the total concentration of amended metals and their tolerance might be lower.

## Discussion

### Geochemistry and metal deposition in creek sediments

Despite physical remediation in the area of the former Ronneburg uranium-mining district, Germany, small drainage creeks are still contaminated with heavy metals due to upwelling contaminated groundwater. Creek sites had a stable pH at 2.7 (site R1) and 6.3 (site R3) over the 1.5 year study period. Unlike what was observed at other AMD sites (Gray, 1998; Sarmiento et al., 2009; Egal et al., 2010), we did not observe seasonal variations in geochemical parameters. This might be caused by the continuous high impact of ground and seepage water inflow at numerous locations to the creeks.

All creek sites had high heavy metal concentrations, with Fe as the predominant metal in waters and sediments. At the slightly acidic site R3 (pH 6.3) heavy metals were highly enriched in the creek sediment compared to the other sites. These metals were mainly bound to the iron oxide fraction, suggesting sedimentation of heavy metals via e.g., co-precipitation or adsorption to iron oxides. Concentrations of Cu, Zn, and Cd in creek water at site R3 were below the drinking water guideline values set by the World Health Organization (WHO, 2008), reducing the impact to downstream ecosystems. The metal concentrations in R3 sediment exceeded the precautionary values for soils as set by German law (μg per g dry wt: Ni, 15–70; Cu, 20–60; Zn, 60–200; Cd, 0.4–1.5; BBodSchV, 1999) and were high compared to other former mining sites with neutral to slightly acidic pH (Willett et al., 1994; Podda et al., 2000; Aykol et al., 2003; Friesl et al., 2006; Rastogi et al., 2010).

The sedimentation of heavy metals with chemically or microbially formed highly abundant iron oxyhydroxides could provide a temporal sink for heavy metals, i.e., retardation, but also a potential source for remobilization, for instance by FeRB. Fe(III) reduction *in situ* could lead to the release of heavy metals, similar to what has been observed for Gessenbach creek bank soil (Burkhardt et al., 2010). An even more stable sink could be provided by metal sulfide precipitation promoted by sulfate-reducing microorganisms (Gadd, 2004), as was demonstrated for nearby creek bank soil (Sitte et al., 2010). However, the relevance of different species for remediation aspects, e.g., natural attenuation, is strongly dependent on their quantities and stability. It should be mentioned that R3 sediment is manually removed from the creek bed from time to time, facilitating long-term removal of heavy metals from the site.

### FeOB and FeRB communities in creek sediments

#### Specificity of the newly designed sideroxydans *spp*.-specific primer set

The specificity of the newly designed primer pair was verified with several different methods. Initially it was tested *in silico* using online probe match databases. The PCR and qPCR assays with the new primers yielded amplicons with DNA of *Sideroxydans* sp. CL21 and ES-1 as a template but not with DNA of other organisms, namely *Gallionella* sp. ES-2, *G*. *ferruginea, Gallionellaceae* strain HDD, *Thiobacillus* sp., *Dechloromonas* sp., *Albidoferax ferrireducens, Xanthomonadaceae, Toluomonas* sp., *Acidocella* sp., *Pleomorphomonas* sp., and *Geobacter* sp. In addition, PCR products amplified from an environmental sample using the newly designed primer pair were cloned and sequenced. BLAST analysis revealed that all of the 33 analyzed cloned sequences were affiliated with the *Sideroxydans* genus with *Sideroxydans* sp. CL21 (98–99% 16S rRNA sequence identity) and *Sideroxydans* sp. ES-1 (97–98% sequence identity) being the nearest and second nearest cultured relative. Thus, we confirmed that the new primer pair is specific for *Sideroxydans* spp.

#### Community diversity

Quantitative PCR results showed that at least 74% (R2) and 78% (R3) of the bacterial communities could be assigned to groups of FeOB or FeRB. In addition, 47% of sequences detected in all clone libraries were closely related (≥97% sequence identity) to cultured FeOB or FeRB. The active community of R3 sediment harbored an even higher fraction of sequences related to FeOB/FeRB (53% in the RNA-derived library) than the total community (18% in the DNA-derived library), suggesting an importance of iron transformation processes. As cloning and qPCR were performed on pooled field samples collected in different years the similar results support our conclusion of the importance of these metabolic groups. Future work using transcriptomics could provide evidence for microbially mediated iron redox reactions. Low diversity was found especially in the moderately acidic R2 creek sediment with 80% of cloned sequences closely related to FeOB/FeRB and within these 69% of sequences related to only one organism, the FeOB *Gallionella ferruginea*. Such a high dominance of a metabolic group is typically only seen in extremely acidic AMD waters; at the Richmond Mine, Iron Mountain, USA, an average of 77% of bacterial and archaeal clones from various samples with a pH of 0.75–1.4 were closely related (≥97% 16S rRNA gene sequence identity) to FeOB (Druschel et al., 2004). Yang et al. (2008) found that 77% of clones from extremely acidic AMD in the Shen-bu copper mine, China, were closely related to FeOB and FeRB, similar to studies in the Lechang Pb/Zn mine (Tan et al., 2007) and the Yunfu sulfide mine, China (He et al., 2007).

#### Presence of FeOB and FeRB

The stable geochemical conditions observed in creek waters and sediments suggest that the microbial community structure is not highly variable in time and thus representative for the sites' geochemistry. Two FeOB and FeRB typically associated with AMD sites were detected, namely “*Ferrovum myxofaciens*” and *Acidiphilium acidophilum*, whereas the extremely acidophilic AMD-known organisms *Leptospirillum ferrooxidans, Acidithiobacillus ferrooxidans*, or *Acidocella* spp. (Hallberg, 2010) were not observed at any of the studied sites. We also did not observe the occurrence of the neutrophilic FeOB *Leptothrix* spp. at the Ronneburg site. Nonetheless, we found sequences related to several other neutrophilic and moderately acidophilic FeOB/FeRB, which were found at contaminated environments before, such as *Gallionella ferruginea, Acidovorax* sp., *Sideroxydans* sp. CL21, *Ferritrophicum radicicola*, and *Geothrix fermentans* (Table 1). Unexpectedly, clone sequences from the extremely acidic site R1 were also related to neutrophilic iron-associated microorganisms like *Gallionella ferruginea, Acidovorax* sp., and *Albidoferax ferrireducens*. The neutrophilic nitrate-reducing FeOB *Acidovorax* sp. strain BrG1 (Straub et al., 2004) represented 24% of clone sequences in the active community of pH 6.3 R3 sediment. Considering that nitrate is present and that there were low but detectable numbers of cultivatable nitrate-reducing FeOB, this suggests that this organism might be coupling iron oxidation to nitrate reduction in the thick anoxic zone of R3 sediment.

#### Abundance of FeOB/FeRB

The relative abundance of FeOB-related 16S rRNA gene sequences was lower in R3 sediment (37%) than in R2 sediment (72%) (Figure 4). However, *Gallionella*-related sequences had similar and very high absolute gene copy numbers in both sediments, and clone libraries and qPCR confirmed also the presence of other microaerobic FeOB, *Sideroxydans* spp. and *Ferritrophicum radicicola*, in R3 sediment. The highest number of cultured anaerobic, nitrate-reducing FeOB was detected in R3 sediment and FeOB such as *Acidovorax* sp. BrG1 were detected in the RNA-based clone library of this sediment. Thus, microbial iron oxidation might be important *in situ* in the microoxic and anoxic zones of the thick and most reduced R3 sediment with varying redox potentials despite the higher pH of 6.3. The more reduced conditions appeared to favor the occurrence of FeRB in R3 sediment compared to R2 sediment, as demonstrated by higher absolute and relative (41%) FeRB-related 16S rRNA gene copy numbers as well as a high number of *Albidoferax ferrireducens*-related sequences in the RNA-based clone library.

To investigate differences in the iron redox active microbial communities under moderately and slightly acidic conditions, we determined the abundance of different FeOB and FeRB, which are known to have a different pH growth range (Table 1). The acidophiles “*Ferrovum myxofaciens*” and *Acidiphilium* spp. had higher absolute and relative 16S rRNA gene copy numbers at site R2 (pH 4.4) than at site R3 (pH 6.3), as expected. Overall these groups comprised only a small part of the bacterial communities in R2 and R3 sediment based on qPCR, which was supported by the clone library results from a different time point. Clone library results also indicated that they were more important in R1 sediment (pH 2.7). *Sideroxydans* spp. appeared to be more abundant in R2 sediment, possibly because they might be mostly related to the strain CL21 (as detected by sediment clone libraries and the *Sideroxydans*-specific PCR clone library), which has a pH range of 4–6 (Lüdecke et al., 2010). Nonetheless, *Gallionella* spp. did not increase in abundance from pH 4.4–pH 6.3, as would have been expected from the known pH growth range of 5–7.6 (Hallbeck and Pedersen, 1990). It had similar and very high absolute and relative gene copy numbers in both sediment communities and was also detected in clone libraries of all three sites. This suggests the existence of *Gallionella* strains capable of growth at lower pH than currently known, as was also observed at other AMD sites (for references, see Table 1). *Gallionella*-like stalks were observed at site R3 but also at site R2, which was surprising as it was reported to not start stalk-formation below pH 6 (Hallbeck and Pedersen, 1990). A possible explanation for the high abundance of *Gallionella* spp. and *Sideroxydans* spp. at site R2 might be the ideal geochemical conditions due to a nearby inflow of microoxic and iron-rich groundwater from a drainage pipe. As *Gallionella* spp. were highly abundant in R3 sediment, which had the highest heavy metal contamination of all three sites, these microaerobic FeOB might have high heavy metal tolerances.

### Metal tolerance of microaerobic FeOB enrichments

Enriched indigenous FeOB from the highest metal-contaminated R3 sediment tolerated metal concentrations much higher than concentrations observed in creek water or sediment. Growth was observed in the presence of 35 mM Co, 24 mM Ni, and 1.3 mM Cd (dissolved metal concentrations). Dissolved concentrations are the most biologically relevant as those are what would require up-regulation of metal resistance systems (Nies, 1999). The observed tolerated concentrations were much higher than the minimal inhibitory concentrations reported for *Escherichia coli* (1.0 mM Co, 1 mM Ni, and 0.5 mM Cd, Nies, 1999) or the concentrations reported for enriched FeRB from nearby Gessenbach creek bank soils (Burkhardt et al., 2011). Such a high metal tolerance of the indigenous FeOB provides support for their metabolic activity *in situ*.

## Author contributions

Study conception and design: Kirsten Küsel, Denise M. Akob, Maria Fabisch. Administrative support: Kirsten Küsel, Denise M. Akob. Collection and assembly of data: Maria Fabisch, Felix Beulig. Data analysis: Maria Fabisch, Felix Beulig, Denise M. Akob. Data interpretation: Maria Fabisch, Felix Beulig, Denise M. Akob, Kirsten Küsel. Manuscript drafting: Maria Fabisch, Denise M. Akob, Kirsten Küsel. Critical revisions to the manuscript: Maria Fabisch, Felix Beulig, Denise M. Akob, Kirsten Küsel.

### Conflict of interest statement

The authors declare that the research was conducted in the absence of any commercial or financial relationships that could be construed as a potential conflict of interest.
