# Supplementary material for: Surprising abundance of Gallionella-related iron oxidizers in creek sediments at pH 4.4 or at high heavy metal concentrations
Source: Front Microbiol. 2013 Dec 18;4:390. doi: 10.3389/fmicb.2013.00390 (PMC3866512; doi:10.3389/fmicb.2013.00390)
Supplement: Supplementary file 1 [file DataSheet1.PDF]

Supplementary Material

Surprising abundance of *Gallionella*-related iron oxidizers in creek sediments at pH 4.4 or at high heavy metal concentrations

Maria Fabisch[1], Felix Beulig[1], Denise M. Akob[1,2] and Kirsten Küsel[1]*

[1] Aquatic Geomicrobiology Group, Institute of Ecology, Friedrich Schiller University Jena, Jena, Germany.
[2] U.S. Geological Survey, National Research Program, Reston, VA, USA.

*** Correspondence:**
Prof. Dr. Kirsten Küsel
Friedrich Schiller University Jena
Institute of Ecology
Aquatic Geomicrobiology Group
Dornburger Strasse 159
D-07743 Jena, Germany
kirsten.kuesel@uni-jena.de

Running title: *Gallionella* in acidic creek sediments

1. Supplemental Tables

Supplemental Table S1. Metal concentrations and growth in gradient tubes for metal tolerant iron oxidizer enrichments.

Metal treatment	Concentration added (mM)	Dissolved concentration measured (mM)[a]	Growth
Co	0.5	0.12	yes
	1.0	0.21	yes
	5.0	2.35	yes
	10.0	5.15	yes
	50.0	34.45	yes
Ni	0.5	0.11	yes
	1.0	0.08	yes
	5.0	0.49	yes
	10.0	1.63	yes
	50.0	24.01	yes
Cd	0.5	0.23	yes
	1.0	0.13	yes
	5.0	0.72	yes
	10.0	1.32	yes
	50.0	13.90	no

[a] Values are the concentrations measured by ICP-MS in uninoculated controls.

Supplemental Table S2. Statistical analyses of 16S rRNA (gene) clone libraries of creek sediment from sites R1 (DNA-R1), R2 (DNA-R2), and R3 (DNA-R3, derived from community DNA extract; and RNA-R3, derived from community RNA extract).

Site	Library	No. of clones	No. of OTUs	Coverage
R1	DNA-R1	84	27	87%
R2	DNA-R2	86	19	84%
R3	DNA-R3	67	34	63%
	RNA-R3	74	30	78%

OTU, operational taxonomic unit.

2. Supplemental Figures

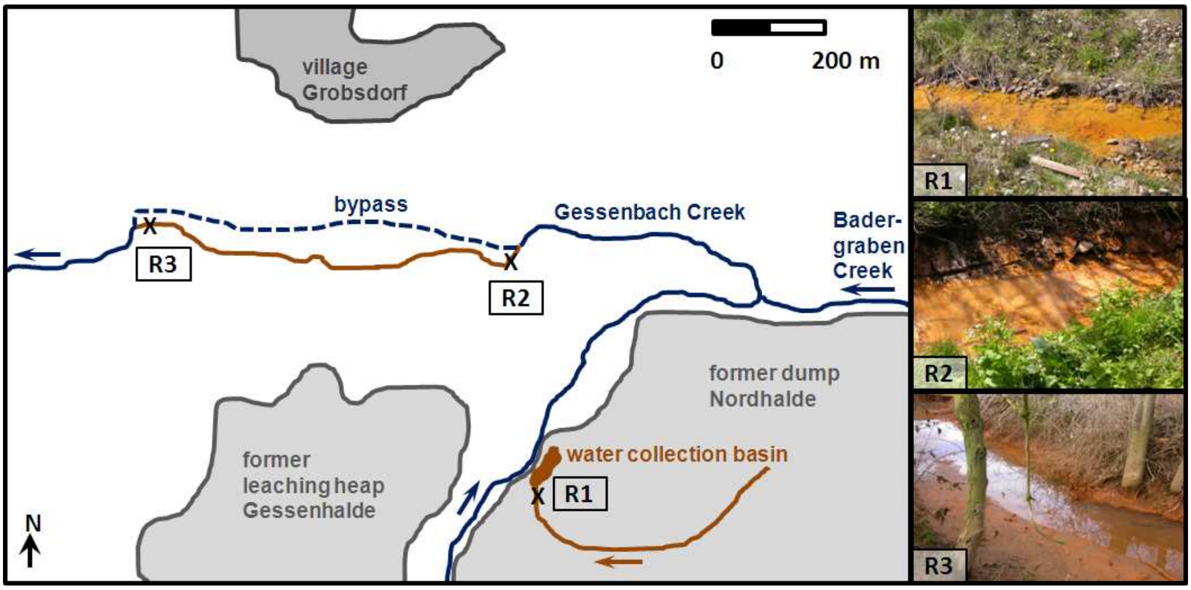

Supplemental Figure S1. Schematic map and photographs of the field sites R1, R2, and R3, which are located in the former Ronneburg uranium-mining area (Thuringia, Germany; locations E 4510990/ N 5635353 site R1, E 4510874/ N 5635777 site R2, E 4510271/ N 5635859 site R3, Gauss-Krueger Potsdam coordinate system). Site R1 is located at the afflux of a drainage creek to a seepage water collection basin, whereas sites R2 and R3 are situated in the former bed of the Gessenbach creek, which was bypassed around this contaminated area. Contaminated sectors of the creeks are drawn in brown instead of blue in the map. Note the rust-orange color of the creek sediments in the photographs.

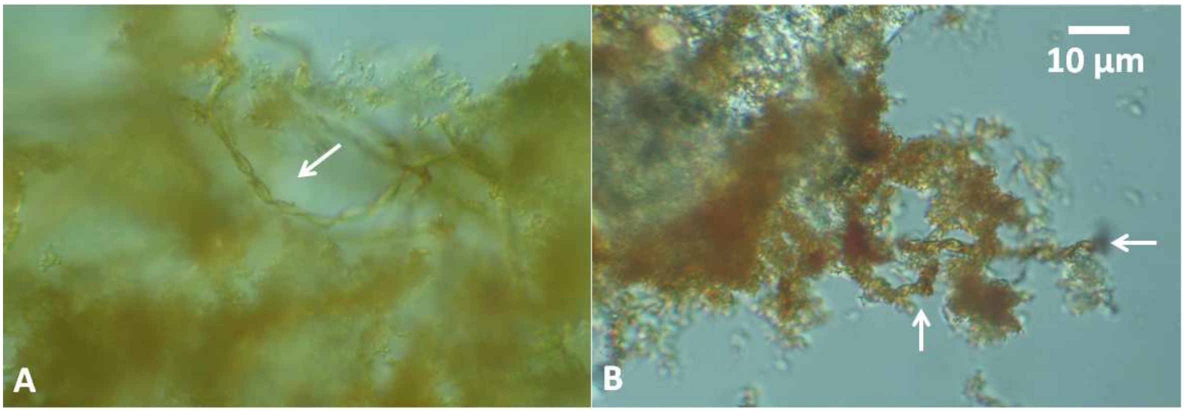

Supplemental Figure S2. Representative light micrographs of *Gallionella*-like stalks associated to iron oxyhydroxides on glass slides after one week of incubation in creek water of site A) R2 or B) R3.
